# A Darwinian and Physical Look at Stem Cell Biology Helps Understanding the Role of Stochasticity in Development

**DOI:** 10.3389/fcell.2020.00659

**Published:** 2020-07-23

**Authors:** Jean-Pascal Capp, Bertrand Laforge

**Affiliations:** ^1^Toulouse Biotechnology Institute, University of Toulouse, INSA, CNRS, INRAE, Toulouse, France; ^2^LPNHE, UMR 7585, Sorbonne Université, CNRS/IN2P3, Université de Paris, Paris, France

**Keywords:** stochastic gene expression, non-genetic heterogeneity, pluripotency, multipotency, biological organization, emergence, constraints, ontophylogenesis

## Abstract

Single-cell analysis allows biologists to gain huge insight into cell differentiation and tissue structuration. Randomness of differentiation, both *in vitro* and *in vivo*, of pluripotent (multipotent) stem cells is now demonstrated to be mainly based on stochastic gene expression. Nevertheless, it remains necessary to incorporate this inherent stochasticity of developmental processes within a coherent scheme. We argue here that the theory called ontophylogenesis is more relevant and better fits with experimental data than alternative theories which have been suggested based on the notions of self-organization and attractor states. The ontophylogenesis theory considers the generation of a differentiated state as a constrained random process: randomness is provided by the stochastic dynamics of biochemical reactions while the environmental constraints, including cell inner structures and cell-cell interactions, drive the system toward a stabilized state of equilibrium. In this conception, biological organization during development can be seen as the result of multiscale constraints produced by the dynamical organization of the biological system which retroacts on the stochastic dynamics at lower scales. This scheme makes it possible to really understand how the generation of reproducible structures at higher organization levels can be fully compatible with probabilistic behavior at the lower levels. It is compatible with the second law of thermodynamics but allows the overtaking of the limitations exhibited by models only based on entropy exchanges which cannot cope with the description nor the dynamics of the mesoscopic and macroscopic organization of biological systems.

## Introduction

While pioneering works early suggested its preeminent role in cell differentiation, the notion of stochasticity has been increasingly considered in developmental and stem cell biology only since a decade, thanks to advances in single-cell analyses [see among others (Hayashi et al., 2008; MacArthur et al., 2012; Abranches et al., 2014; Mojtahedi et al., 2016; Moris et al., 2016; Richard et al., 2016; Moussy et al., 2017; Semrau et al., 2017; Stumpf et al., 2017)]. Both the inherent probabilistic behavior of embryonic and adult stem cells related to their highly stochastic gene expression (SGE), and the subsequent randomness of differentiation events, encourage to make this stochasticity a key cellular property on which a modern theory of development should be based. Some attempts have been made to introduce mechanisms explaining SGE from the chaotic behavior of a presupposed underlying gene network in the cells. Using the ordinary differential equation (ODE) formalism, the notion of attractor is exhibited, which coupled with Waddington’s idea of canalization, provides a framework increasingly quoted in the literature. Nevertheless, this approach where SGE results from a chaos-induced noise over a purely deterministic inner cell network has its own inner auto-coherence issues (Kupiec, 2009). On the opposite, both biological and physical considerations allow arguing for a theory that considers the acquisition of differentiated features as the result of decreased cellular stochasticity linked to the appearance of dynamically evolving environmental constraints acting at the cell (through compartmentation and mesoscopic structures such as chromatin, nuclear membrane, etc.), tissue (through cell-cell interactions and communications) and organism (through endocrine, immunity and blood networks) levels. In first approximation, these constraints can be defined as the external conditions that exert on a system. Nevertheless, besides this internal/external dichotomy, in an extended system, local dynamics inside the system can also constitute constraints (see below). Here, these constraints take place concomitantly to the stochastic appearance of cellular phenotypes during developmental processes, and cannot be envisaged independently of cellular behavior. The intertwined and reciprocal influences of both cellular stochasticity and environmental constraints can account for the emergence of developing structures in multicellular organisms and provide a paradigm which is fully compatible with physical and chemical laws at molecular and mesoscopic scales. Importantly, this approach provides an easy explanation of cell reprogramming and cell pluripotency capabilities.

## Transcriptional Heterogeneity in Stem Cells

### Molecular Basis of Transcriptional Heterogeneity in Embryonic Stem Cells

Following the establishment of the first embryonic stem cells (ESC) lines in 1998 (Thomson et al., 1998), a decade of works led to characterization of the ESC state (Young, 2011). Especially the pluripotency network centered on the three pivotal transcription factors (TF) Oct4, Nanog and Sox2 has been defined (Orkin and Hochedlinger, 2011; Li and Izpisua Belmonte, 2018). First, these factors act to positively regulate their own promoters creating an interconnected autoregulatory loop. Second, they occupy and activate the expression of genes necessary to maintain the ESC state, and contribute to repress genes encoding lineage-specific TF. Thus they prevent exit from the pluripotent state (Young, 2011).

Moreover ESC possess an unusual nuclear structure where DNA is arranged in a less compacted chromatin structure compared to differentiated cells. This is associated with the rapid turnover of chromatin proteins which is a hallmark of weak interactions with DNA (Meshorer et al., 2006). Accordingly, ESC are enriched in epigenetic marks associated with elevated gene expression, and possess less marks that compact chromatin enough to prevent any gene expression (Spivakov and Fisher, 2007). This enrichment has later been linked to widespread, generalized, stochastic and pervasive gene expression in ESC (Efroni et al., 2008), with most of the tissue-specific genes analyzed being sporadically expressed at low level while they were not expected in ESC. On the opposite, a large-scale repression of gene expression occurs during differentiation with expression profiles becoming more specific and stable in differentiated cells. Thus stem cells cannot be defined as a cell type characterized by stable phenotypes. They harbor a permanent unstable state and do not stably express specific genes.

Some TF considered to be specific of ESC, such as Nanog (Chambers et al., 2007) and others (Hayashi et al., 2008) involved in pluripotency are expressed with high heterogeneity from cell-to-cell. Interestingly, the propensity to differentiate depends on their expression levels (Kalmar et al., 2009; Radzisheuskaya et al., 2013). Especially, mouse ESC expressing Nanog at a lower level can differentiate more easily and can be considered as being in a “primed” state (Abranches et al., 2014). Cells expressing Nanog at higher levels possess more stable gene expression profiles in various growth conditions, and a minimal expression of differentiation genes (Abranches et al., 2014). Nevertheless, ESC populations cannot be simply divided into two distinct subpopulations with higher and lower levels of pluripotency factors. There is a continuum of states from high to low pluripotency (Klein et al., 2015). Exit from pluripotency occurs only through a continuum of intermediary states and not as an abrupt transition toward a new state (Hough et al., 2014). Thus pluripotency appears to be more a state of dynamic heterogeneity of a population driven by transcriptional noise than a discrete state dependent on the fixed expression of a small set of genes (Kalmar et al., 2009). Moreover the function of the gene regulatory network centered around Nanog might be to control this dynamic heterogeneity (Kalmar et al., 2009).

### Transcriptional Heterogeneity in Adult Multipotent Stem Cells

Generalized transcription activity was already observed in hematopoietic stem cells (HSC) in the early 2000s when terminal differentiation genes were shown to be present before any commitment, with a large part of the genome expressed in a less differentiated state (Terskikh et al., 2003). This phenomenon has been later studied by the most powerful tools in molecular and cellular biology which confirmed that differentiation of hematopoietic cells is clearly probabilistic (Hume, 2000; Chang et al., 2008; Pina et al., 2012). The pioneering work led by Sui Huang on progenitors of the hematopoietic system led to two major observations (Chang et al., 2008). On the one hand, these cells heterogeneously express Sca-1 which is associated with HSC, and when a subpopulation with homogenous Sca1 levels is isolated and regrown, the initial heterogeneity is restored upon a few days. On the other hand, the Sca1 level is linked to the propensity to differentiate, reflecting the important bias associated with its expression.

More molecular details about the phenomena beyond the stochastic behavior of HSC were obtained when other authors observed that different regulators of HSC differentiation vary independently and stochastically in these multipotent cells, what has been linked to priming toward different cell types (Pina et al., 2012; Moignard et al., 2015; Moussy et al., 2017; Giladi et al., 2018). For instance, the erythroid lineage is primed while many markers of other lineages are also expressed (Pina et al., 2012). During cell fate specification of haematopoietic multipotential progenitor cells, mixed-lineage intermediates with concurrent expression of haematopoietic stem cell/progenitor and myeloid progenitor TF seem to be mandatory (Olsson et al., 2016). These results showed that there is no coordination in the HSC expression pattern. Bone marrow differentiation is by far less hierarchical than previously assumed (Giladi et al., 2018). The early differentiation steps occur sporadically by the stochastic expression of differentiation genes in individual cells (Muers, 2012). Study of the early differentiation of T cells led to the same conclusion that, contrary to the expression of only one set of lineage-specific TF, there is an ubiquitous high-level co-expression of antagonistic TF in individual cells (Fang et al., 2013). Moreover, cytokines, which are generally correlated to specific lineage, are stochastically expressed in small subpopulations without being associated to specific TF (Fang et al., 2013). More generally, genes are continuously repurposed across lineages and fates, bringing single cells from different lineages closer in transcriptional space and skewing models for differentiation dynamics (Giladi et al., 2018).

The first analysis at the single-cell scale of the development of an entire mammalian organ was performed on the hematopoietic system (Moignard et al., 2015). By studying the early development of blood cells in mice, it was shown that the early differentiation of HSC is asynchronous, with cells at multiple stages expressing similar combinations of developmental regulators. There is a continuous emergence of blood-specified cells underlying an increase in the proportion of cells expressing a given gene between stages. Graded expression changes over time are not consistent with a discrete switch at a specific developmental moment. This work showed that cells destined to become blood and endothelial cells arise at all stages of the analyzed time course rather than in a synchronized fashion at one precise moment.

Most of these works observed and acknowledged the existence of heterogeneity in cell differentiation, but still argued for a deterministic model with a certain degree of noise. Nevertheless, the best illustration of the dynamic nature and variable timing of cell fate commitment of HSC was provided by Moussy et al. (2017) and led to the conclusion that cell fate decision is a dynamic and complex process characterized by fluctuations that are far from the simple deterministic and binary switches between opposite options that are usually envisioned.

### Necessity for a “Statistical Mechanics” of Pluripotency

What these works performed in the last decade revealed is at the opposite of the model predominant in the early 2000s where stem cells were supposed to be finely regulated to homogeneously express only few genes allowing pluripotency and self-renewal, and to homogeneously react to differentiation cues. Only few researchers early highlighted the fact, mainly based on the hematopoietic system, that it seems indeed impossible to define a molecular “signature” of stem cells and that generalized and variable gene expression is what defined stemness (Zipori, 2004). From this perspective, one can argue that differentiation is above all the suppression of this widespread SGE (Efroni et al., 2009) and even propose a model where it is highly determined by the transition of the chromatin organization from a dynamic and open state to a more stable and closed state (Ram and Meshorer, 2009).

In the light of these results revealing the importance of randomness in the stem cell behavior, pioneering works by Paldi suggested the central role of the chromatin in the suppression of stochasticity and proposed an explicit relationship between metabolism and the level of cellular variations (Paldi, 2003). MacArthur and Lemischka (2013) proposed in 2013 to apply the concepts of statistical mechanics to pluripotency. Their main idea is that pluripotency is not a property of a cell but the property of a cell population which can consequently be analyzed using the concept of entropy that measures how the different patterns of genetic expression are distributed among the space of all possible states. The equilibrium between states is thought to be ergodic, i.e., recovered very quickly when disturbed, so that every single cell experiences quickly a vast fraction of the possible expression patterns. This allows explaining how quickly an erased phenotypic part of the population can be recovered among cells as observed in different experiments. The permissive chromatin in stem cells imposes weak constraints on gene expression and makes impossible the definition of the pluripotent state at the single-cell level. Functional pluripotency emerges from the dynamic variability of the pluripotent states (MacArthur and Lemischka, 2013).

In the view of the authors, the equilibrium between states is still an intrinsic property of the cell through its genetic regulation network and stochastic microscopic events are seen as a noise over an intrinsically determined cell dynamics regulating the equilibrium between states. Although not always acknowledged by researchers using stochastic networks to describe the differentiation of ESC who would not entertain noise superimposed over a deterministic system, this hypothesis made to save genetic determinism is unfortunately introducing an incoherence in the model since it does not explain how external constraints have to be integrated in the picture. From their point of view, the primed cells in a specific state of the pluripotency pool of states are then determined to be able to respond to specific external signals. In this approach, the integration of those signals by the cell is not included in the model to explain how they can change the equilibrium of states and restrict the number of possible states. This remark also raises the question of the number of accessible states to a cell so that the process remains ergodic. Are the number of states really reduced or is it only their probability of appearance which is modified so that many states become unlikely to actually form? An elegant way to solve this issue is to assume that cumulative constraints from the inner cell structure (chromatin and compartments) is always strong enough so that the space of genetic patterns is always small enough for the equilibrium between states to remain ergodic. Complementary, the regulation of the equilibrium should not only be intrinsic to the cells to accommodate the so-called external signals. The dynamics of the chromatin organization, which is the main regulator of the accessible states equilibrium, should be allowed to depend on larger scale constraints at tissue or organism level, i.e., not only to cell level constraints.

Consequently, this dynamic variability should be spatiotemporally regulated *in vivo* while these restrictions would be largely released *in vitro* and the intrinsic variability in the population should become apparent depending on culture conditions as seen experimentally (MacArthur and Lemischka, 2013). Thus, one can assume that these stochastic expression variations give rise to a state of dynamic equilibrium in which single cells transit randomly between distinct metastable states, and these highly variable and high-entropy expression patterns are likely to be responsible and necessary for the large number of possible developmental commitments (MacArthur and Lemischka, 2013). Transcriptional heterogeneity in stem cells is thus considered as an advantage for diversifying phenotypes in populations that require diverse potentialities (Torres-Padilla and Chambers, 2014). Then constraints, established when differentiation progresses, stabilize some phenotypes and decrease population entropy because chromatin acquires a less permissive state and expression patterns become more tightly constrained.

## Stochastic Differentiation Processes

### Probabilistic Differentiation

Until recently, cellular differentiation was widely considered as deterministically induced by microenvironmental signals. Nevertheless, stochastic “choices” between several lineages are now demonstrated in many cases, without any role of inducing signals. For instance, in the HSC niche, the less differentiated cells are a mixed population of already primed cells (Yamamoto et al., 2013). This study shows that priming is not the result of niche factors guiding HSC differentiation. Instead, it is largely generated by intrinsic cellular factors (Yamamoto et al., 2013). Also, the fate of individual B cells following activation leading to their differentiation into diverse populations depends on the competition between intracellular processes varying stochastically (Duffy et al., 2012). These variations explain how lymphocytes produce the different terminal populations in reproducible proportions (Duffy et al., 2012). The probabilistic distribution does not result from specific instructions received by individual cells but from collective behavior in response to environmental cues, what could be generalized for differentiation processes from embryos to the hematopoietic system (Duffy et al., 2012).

Other developmental phenomena beginning with a probabilistic choice followed by microenvironmental stabilization are the formation of the retinal mosaic (Wernet et al., 2006), the acquisition of neuronal identity (Lesch et al., 2009) or the intestinal cell differentiation (Raj et al., 2010). Adult stem cells seem to differentiate because of stochastic intracellular phenomena rather than extracellular regulation. Environmental factors may only bias the internal stochastic processes (Wabik and Jones, 2015). This can be interpreted as a selective phenomenon among phenotypes that randomly appeared. Indeed, it is proposed that the varied expression of genes associated with each alternative fate in single cells can act as a substrate for selection by signals (Moris et al., 2016). An example is provided by Guye et al. (2016) who showed that cell fate decisions occur as a stochastic switch integrating cell-autonomous (here Gata6 levels that randomly fluctuate) and non-autonomous (cell types nearby) parameters. Only cells expressing by chance a given level of protein in the adequate environmental context start differentiating. This proposal has been largely developed in a theoretical model of cell differentiation based on Darwinian selection called ontophylogenesis (Kupiec, 1996, 1997, 2009) (see below).

Lineage tracing techniques also revealed that adult stem cells have a far larger differentiation potential when they are out of their initial tissue environment and transplanted in other locations (Goodell et al., 2015). These observations suggest that cell fate is not irreversibly predestined and that the microenvironment where these heterogeneous cells reside restrains lineage choice (Goodell et al., 2015). Differentiating cells are clearly not hierarchically organized and differentiation pathways are not unidirectional (Zipori, 2005; Knaan-Shanzer et al., 2008; Tetteh et al., 2015). An uncertainty exists in terms of origin and fate for each cell, together with variability in the timing and pathway toward differentiation for cells acquiring the same phenotypes. This suggests that stochasticity in differentiation processes finds its origin in the stochastic appearance of differentiation features.

### Modulation of Stochastic Gene Expression During Differentiation

Recent works now give evidence that a degree of coordination of SGE is modulated during development by cell-cell communication. The canonical example is provided by the phenomena underlying fate decisions in the inner cell mass (ICM) of the blastocyst stage embryo into the epiblast (EPI) and primitive endoderm (PE) lineages [for review, see Simon et al. (2018)]. Single-cell transcriptomics analyses revealed that cells of the preimplantation mouse embryo uncover considerable transcriptional heterogeneity of ICM cells at the 32-cell stage (Guo et al., 2010; Ohnishi et al., 2014). Following this initial phase of stochastic expression of individual genes, FGF/ERK signaling interfaces with the Nanog and Gata6 TF to ensure cell differentiation in the ICM (Simon et al., 2018). Signaling and SGE are interconnected and influence one-another, and cell communication exploits and buffers noise to enable formation of the tissue with reproducible proportions of EPI and PE cell types (Simon et al., 2018). For a physical perspective, signals can be seen as constraints on the cell, a feedback of the tissue. In that perspective, organogenesis and cell differentiation are two folds of a single multiscale process.

Other convincing examples of reduction of SGE and progressive coordination of gene expression profiles from cell-to-cell in tissues during development appeared in the last years (Featherstone et al., 2011, 2016; Ji et al., 2013; Pelaez et al., 2015). For instance, when cell differentiation is tracked *in vivo* during development of the pituitary tissue in transgenic rats in which luciferase was expressed from the prolactin (a pituitary hormone) promoter (Featherstone et al., 2011), it appeared that the first endocrine cells showed highly pulsatile expression which is then stabilized as tissue develops and cell number increases. The pulsative and heterogeneous luminescence was restored when cells were dissociated, showing that the stabilized expression pattern depends on the tissue architecture and cellular interactions. More precisely, direct cell contacts involving gap junctions ensure this coordination of prolactin gene expression because trypsin-mediated digestion of extracellular proteins or pharmacological inhibition of intercellular gap junctions reduced transcriptional coordination between cells (Featherstone et al., 2016). Thus, cell communication reduces SGE and phenotypic heterogeneity among differentiating cells.

Finally, only recent multiscale analyses of differentiation showed that it must be understood as a release of previous constraints that maintained SGE at relative lower levels in progenitors cells, followed by peak of variability that occurs before a reduction of SGE at lower levels than initially because new constraints are applied (Richard et al., 2016; Moussy et al., 2017). Indeed, while gene expression is initially widespread and highly variable when growth conditions ensuring the maintenance of the stem state are released, cells progressively transit toward more homogeneous, coordinated and restricted gene expression profiles (Efroni et al., 2008; Richard et al., 2016; Moussy et al., 2017). This cellular entropy appears to decrease from this transient unstable state with the highest SGE to the final stable differentiated state obtained under new environmental constraints (Richard et al., 2016). This trend is observed in single-cell transcriptomic data sets from various cell differentiation systems that have been recently studied in a more systematic way (Gao et al., 2020). Thus cell fate decision appears to be a process of spontaneous variation and selective stabilization in which each cell randomly explores many different possibilities by expressing a large variety of genes before finding a stable combination corresponding to the actual environment (Moussy et al., 2017). SGE and environmental constraints concomitantly act to produce stabilized phenotypes. This is in remarkable agreement with earlier theoretical predictions, especially in the ontophylogenesis theory. Nevertheless, this interplay has been diversely interpreted in different models of cell differentiation.

## Models of Cell Differentiation Based on Stochastic Gene Expression

SGE is now recognized as a driving force for diversifying phenotypes in undifferentiated cell populations, and as a responsible for lineage priming in stem cells. Alternative theories of development are needed to take into account this initial phenotypic plasticity, and its progressive reduction producing stable differentiated cell types. Especially, several models of cell differentiation have been formulated in which a peak in the gene expression variability is expected to occur.

### Attractors and Self-Organization

The epigenetic landscape proposed by Conrad Waddington more than 60 years ago (Slack, 2002) has gained lots of attention in the last decade because it metaphorically describes the cell “canalization” toward differentiation. In this scheme, cells look like balls rolling in a landscape made of hills and valleys, until a final point of minimal altitude. Each valley corresponds to a precise lineage differentiation pathway, and gravity in the driving force leading to terminal differentiated types. But what is the biological reality corresponding to the gravitational force in Waddington’s landscape? Why do cells roll toward points of minimal altitudes corresponding to stable cell types?

Several authors have used the dynamical systems theory to reintroduce the concept of attractor states first proposed by Stuart Kauffman decades ago (Kauffman, 1969). Sui Huang especially considers the stem cell state and differentiated states as attractor states created by the global architecture of the gene regulatory network (GRN) which orchestrates gene activities to produce the variety of stable cellular states (Huang, 2011). In this framework, state transitions, such as differentiation of a stem cell, arise when varying environmental conditions together with the action of SGE destabilize the current attractor state enough to generate the transition toward another attracting state in the GRN (Huang et al., 2009; Mojtahedi et al., 2016). Only regulatory signals through activation of alternative signal transduction pathways and/or highly stochastic fluctuations of gene expression levels can change enough the expression of a specific set of genes of the network and produce state transition. In this model, cell communications act to coordinate state transitions and to ensure the “correct” canalization of the various cell types in tissues but attractors are fundamentally self-organizing and self-stabilizing states leading to stable gene expression profiles. Moreover, these states are particularly robust to stochastic expression variations of individual genes. Random fluctuations in gene expression are only rarely able to trigger state transition.

Kunihiko Kaneko also employed the concept of attractor state and Waddington’s epigenetic landscape to explain stable differentiation states, but insisted on the necessity to intimately couple both SGE and cell-cell interactions for modeling cell differentiation (Furusawa and Kaneko, 2012). Especially, cell interactions with other differentiating or differentiated cells both stabilize the attractor state and decrease SGE as cells become more differentiated, so that the range of states that the cell could visit decreased with the developmental course (Kaneko, 2011). This synchronization explains the influence of cell interactions in maintaining a low level of protein variations among differentiated cells, but this decrease is only a collateral effect of the attractor state that remains the driving force for differentiation.

In the Kaufmann and Huang’s attractor model, gene expression profiles self-organize toward attractor states because these states are “encoded” in the GRN. Instructive signals produced by extracellular entities are needed to provoke destabilization and state transition. Natural selection during evolution would have conserved only optimized developmental trajectories in the landscape, especially to prevent attracted cells from changing direction toward other attractors without destabilizing signals. But this concept is not a real alternative to the genetic program because one can consider that the attractor states virtually present in the GRN constitute a new form of finalist explanation in development similar to the genetic program. Indeed, one can hardly argue that these attractors are not conceived as final states encoded in the genome, even if they are acquired through a self-organizing and self-stabilizing process.

Moreover, in the Huang/Kauffman model, the valleys (the attractors) guide (canalize) cells, each valley corresponding to a phenotype. However, it is now shown that cells fluctuate between two phenotypes before stabilizing (Moussy et al., 2017). Thus they “jump” from one valley to another before stabilizing, a phenomenon that was previously predicted (Kupiec, 1996) and modeled (Laforge et al., 2005). This observation is in clear contradiction with the attractor model based on Waddington’s canalization: valleys cannot be conceived as canalizing cells if cells can jump from one to the other. A true non-finalist model would need a selective extracellular agent acting among phenotypes stochastically generated by SGE. Kaneko introduces this external element but his model still integrates the attractor concept to explain why the ball rolls down along the Waddington’s landscape.

Historically, the origin of the self-organization theory is the idea that a system can spontaneously organize which is meant by the prefix “self.” Generally, for this reason, the proponents of self-organization have rejected or overtaken Darwinism which on the contrary implies that organization is driven by an external constraint, i.e., natural selection by the environment. Finally, self-organization has never questioned the model of genetic regulation from Jacob and Monod: the GRN relies on such regulations. It assumes that small fluctuations can produce switches between multistable states, but these states are attractor states determined by stable gene networks. In such an approach, the multiscale constraints have no power to change the number nor the properties of the attractor states but only help to reduce the formation of a fraction of those states. Consequently, these models do not predict the emergence of novelty inside the cell just like genetic determinism and do not provide a way of thinking of an intrinsic evolution of species due to stochastic processes inside cells (Kupiec, 2009). Small fluctuations can initiate the switching process but they are then followed by deterministic evolution that can be modeled by deterministic differential equations. Thus the ontology of self-organization is intrinsic stability as the default state. That is why self-organization theorists still consider that SGE is only noise.

### Ontophylogenesis

The ontophylogenesis theory has been originally formulated in 1983 by Kupiec following the main idea that stochasticity of biochemical reactions could play a major role in the cellular differentiation process at a time when this process was mainly thought in terms of genetic program (Kupiec, 1983). It considers cell differentiation as a variation/selection process analogous to evolution (Kupiec, 1996, 1997, 2009). Variations are created by stochastic fluctuations of gene expression, and some patterns are selectively stabilized through interactions with the environment and neighboring cells so that they can be a positive force to create patterning in development see for instance Guye et al. (2016). Thus, it is clearly based on another basis compared to the models mentioned above, a chance-selection principle. This theory is suited to take into account initial stochastic and widespread gene expression and its progressive restriction during differentiation, but without any instructive or finalist aspect.

The Darwinian theory previously broke with the postulate of species stability by putting variation as the *primum movens* for the evolution of species. In the context of cell differentiation, ontophylogenesis makes a step further by also considering variability as the default state at the cell level. Stability is then an optical illusion resulting from a state of equilibrium at a given moment. What appears to be a stable state is the state of an intrinsic dynamical system stabilized transiently by the multiscale constraints that act on the cell fate from the inner cellular level to the external environment including of course the structures at all scales in the organism.

Ontophylogenesis has been extensively described with both theoretical and experimental arguments in articles and books by its author (Kupiec, 1983, 1996, 1997, 2009). In a few words, the stochastic nature of molecular interactions and gene expression makes the appearance of phenotypes, especially differentiation features, a probabilistic phenomenon. This phenotypic heterogeneity would constitute a “substrate” for a selection process by the cellular environment which would stabilize the adequate differentiation or metabolic features in a given environment and at a given time. This environment is constituted by many molecules (diffusible or not) and other cells. Differentiating cells able to interact and communicate with these surrounding molecules and cells are stabilized by cell signaling and post-translational modifications of chromatin proteins which have made the expression possible (Kupiec, 1997). This view is fully compatible with the idea that functional pluripotency is an average property of cells (MacArthur and Lemischka, 2013). As each individual stem cell harbors a variable propensity to be primed because of stochastic expression of key regulators, functional pluripotency can hardly be defined at the single-cell level. But this property ensures that a certain proportion of the cell population has the ability to start differentiating at each time. Again, it is the combination of cell-autonomous (variations in gene expression) and non-autonomous (environmental context) that make priming possible.

Development is conceived as a cellular Darwinian process where gene expression is initially unstable, differentiation features appear stochastically, and cells are selected and stabilized if they express by chance the adequate genes at the right place at the right time to interact with surrounding cells or molecules (Laforge et al., 2005). This model does not exclude a certain degree of intracellular regulation that would repress expression of other differentiation genes when some of them are stabilized for instance to favor canalization (Sunadome et al., 2014) but this mechanism starts by and is not possible without initial stabilization of some genes by cellular interactions. It does not exclude neither that metabolic aspects can be crucial because cells must draw the energy needed to degrade and re-synthesize their components for phenotypic changes (Paldi, 2012). Thus the source of this energy could be a selection factor (a constraint in physicalist terms) which intervenes in the stabilization of certain phenotypes but also in the destabilization of others. Recent works showed that metabolic competition for a resource induces complementary phenotypes and population structure (Varahan et al., 2019).

Finally ontophylogenesis is both the contrary of an instructive mechanism and of a self-organizing phenomenon constrained by GRN. If not stabilized, cells continue to fluctuate or die. Here differentiating cells are also progressively canalized but this canalization is explained by the selective action of the cellular environment, and not by the architecture of the GRN. This dynamic interplay between cellular stochasticity and environmental constraints that act concomitantly and contribute equally to the appearance of stabilized phenotypes paves the way to a better understanding of the causality that explain cell trajectories in multicellular organisms.

Even if the GRN theory has embedded stochastic switches and contains the constraints of signals and cell-to-cell communication, these external constraints only modulate the GRN, they do not alter its structure that remains the essential causal driving force of the system. On the contrary, the main idea behind the ontophylogenesis model is that all organizations at all scales are important for the fate of a given biological system. In that sense, giving a causal role to a structure at a certain scale can be a good effective model but cannot be a fundamental approach of causality. Otherwise, we need to provide an explanation why that specific level is the causal level. More importantly, we need to have a conceptual idea of how that level gets organized. Without this conceptual explanation, we think this is a finalist explanation. The ontophylogenesis model does not have this issue since it takes into account the full diversity of structures that can constrain underlying dynamics. It is the equilibrium between the different levels of organization, which dynamically and reciprocally interact, that accounts for the structuration of the system. Ontophylogenesis is then an extension of Darwinism to ontogenesis.

In this framework, the usual divergence observed between *in vitro* and *in vivo* experimental results finds a natural explanation because all scales have causal importance for the system evolution and structuration. The comparison between experiment and theory becomes more difficult to be done and requires the simultaneous use of *in silico* simulations and *in vivo* measurements of the complex structures of the system under study. When the initial state structures are implemented in a simulation in which the theory is expressed as local relationships between components of the system, one can compare how the real and simulated systems evolve when submitted to the same perturbations.

**FIGURE 1:**
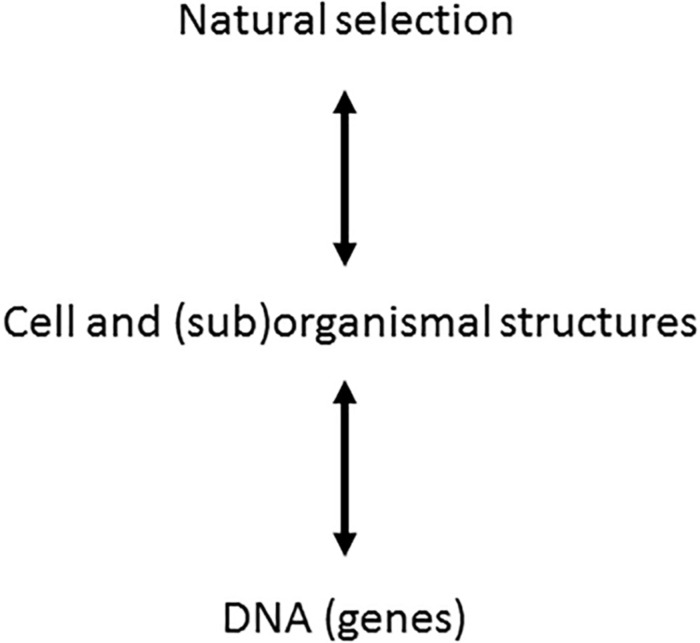
Extension of evolutionary synthesis to ontogenesis. In the frame of the ontophylogenesis model, the relationship between genes and phenotypes is no longer unidirectional but bidirectional. The cell and (sub)organismal structures, subjected to natural selection, sort the molecular interactions, which means that natural selection is a cause of ontogenesis. The genes provide the proteins while the phenotypic structure sorts their interactions from among the possible combinations. The two processes of natural selection and ontogenesis are but one single process of ontophylogenesis [adapted from Kupiec (2009)].

One should notice that ontophylogenesis is consequently not a self-organization theory [see extensive review in Kupiec (2009)]. Indeed the self-organization theory provides organization from a bottom-up perspective where the concept of spontaneous emergence is the main source of generation of new features inside a system. Ontophylogenesis proposes a different perspective which is neither a bottom-up nor a top-down process but both at the same time. Any effective structure retroacts on the other levels of organization, “effective” meaning that the structure results from the constraints coming from both the developmental process and the environment in which the organism is living. For instance, cell-to-cell interactions modulate gene expression probability and alternative editing of RNA at the protein synthesis level while, at the same time, a living being is changing its environment (Levins and Lewontin, 1985). Ontophylogenesis is then indeed a hetero-organization theory providing a satisfactory integration of the holistic and local points of view which, instead of being contradictory, complete each other. This paradigm is illustrated in Figure 1 [adapted from Kupiec (2009)] where we have added a bidirectional arrow between cell and (sub)organismal structure and the natural selection levels since organisms are also structuring their local environment.

Importantly, ontophylogenesis also provides a change of paradigm that reconnects biology to physics and chemistry first principles. Indeed ontophylogenesis is a theory of global order from local disorder on the opposite to all the other mentioned approaches that assume that global order at the scale of an organism derives directly from the local information located in the germinal cell. When epigenetic signaling is similar to the introduction of epicycles on the Aristotelian model of the universe pushing a step forward a level of explanation that escapes to the thought fundamental genetic level, ontophylogenesis offers an alternative giving a paradigm where biological phylogenesis and evolution can be thought coherently. Ontophylogenesis exhibits the importance of the dynamics while biology is usually thought through approaches giving a primordial role to effective structures and functions. These assertions can be enlightened by adopting a physicalist point of view that we propose to now consider.

## Physical Considerations to Cellular Processes and Their Consequences on How Organization of Biological Systems Emerges

### Chaotic Gene Networks Are Not Necessary to Provide Emergence

Understanding from the first principles what is the causality that rules the fate of cells inside an organism is one of the key problems of theoretical biology. The question is of course very difficult since it involves the organization of a complex system with a large variety of components and organized structures that can retroact on the future evolution of a given cell depending on its location in the structured system. So the question also concerns cell collective structure formations and their dynamics.

It is a scientific fact that biological systems at cell scale cannot be modeled using a description in terms of their fundamental constituents at the particle level invoking our current knowledge of the fundamental interactions. Already describing a bound state such as an atom out of its quark and electron content is beyond the scope of the present possibilities. Fortunately, one can build a so-called effective model of the hydrogen atom based on effective components, a proton and an electron to describe its atomic properties such as its emission and absorption spectra. In that specific case, the proton, known to be built from 3 *u* quarks of charge 1/3 each, needs to have a charge +1 to have properties in line with our fundamental understanding of the structure of matter components. Similarly, building effective models of living systems requires the use of fundamental concepts that are coherent with our current knowledge of physics and chemistry laws at low scales, especially if the objective is to provide a broadest and deepest understanding of the biological system organization and dynamics.

Unfortunately, since tens of years now, some of the key concepts used to describe biological systems seem to break this objective. Especially, the concept of information, which is driving theoretical approaches since the beginning of early works of molecular biology, really impacts the way the dynamics of the biological systems is considered. This information concept is broadly understood by biologists as a deterministic path of cell differentiation along the information stored in the DNA. So in that view, the future of the cell inside a body is determined by its gene expression patterns and the dynamics is not so important since the final state is known. This information theory has then a large impact on how biology is overweighting the role of structures in living systems while considering dynamical processes of lower importance since the final state is driven by the information. For instance, looking at embryo development, embryology defines different stages of development characterized by a biological structure while physics would observe the distribution of the times at which the related structure appeared in each embryo by starting a clock at initial time *t* = 0 for each embryo. From the structural point of view, the fact that most embryos develop the related structures provides the impression of a uniform process while the observation of the times at which each structure appears for each embryo is exhibiting the variability of the process producing the structure. One should notice that the information discussed by most biologists is very different from the information theory developed in statistical physics based on the concept of entropy where the level of information in a system is related to the number of its possible microscopic configurations that provides a similar macroscopic state. It is often a subject of misunderstanding between physicists and biologists. In physics, entropy is driven by a very strong principle, the second law of thermodynamics, that states that the entropy of an isolated system can only increase, giving rise to a more and more disordered system. Consequently the increase of order inside an organism requires this system to continuously exchange matter and energy with its environment. Theoretically speaking, it requires to develop a theory of thermodynamics far from the equilibrium. In that perspective, an organism is then a dissipative structure (Prigogine, 1967), i.e., a structure exchanging energy and matter with its external world.

On the opposite side, information in biology is usually related to the deterministic way that will transform a genetic information into a specific structure or function in the organism. The biological paradigm of information is also constraining how the discovery of SGE inside cells is interpreted: the term of “noise” used extensively in the literature generally expresses the idea that this stochasticity occurs on top of a well-defined and information-based behaviors of cells. This denomination forbids thinking stochasticity as a potential driving force of change inside biological systems and as the possible motor of the divergence of living systems as stated by the Darwinian theory of Evolution. In that context, ontophylogenesis suggests that the control of cell stochasticity requires much more than genetic networks (Elowitz et al., 2002), since supracellular structures retroact on the inner cell dynamics.

The observed general difficulty to associate a single gene to a single phenotypic property or biological function and the need of explaining many features with a limited number of genes inside the cells drove genetics into the paradigm of inter-relations between genes to provide a vast number of phenotypic states as a solution to this problem. This idea leads to the definition of gene networks dynamically structured to provide different differentiated states to cells (Emmert-Streib et al., 2014). One should notice that this point of view is just a generalization of the one gene – one molecule – one function paradigm in which the larger complexity is only coming from the combinatorics of several genes. The difficulty of that vision is that a gene network is not well-suited to describe how the cell environment can influence its fate as shown by experimental data. So, one usually envisages the change of a cell state as an intrinsic internal instability (chaotic behavior of a non-linear differential equation system) or as an external event which is very difficult to plug on the gene network from a theoretical point of view. Indeed, a gene network expresses the relationships between the expression patterns of the various genes inside the cells. It describes how an either positive or negative retroaction of each gene expression can modulate the expression of the other genes.

This conception raises the question of how the external constraints and external conditions such as temperature, pressure, chemical concentrations in the tissue can be included in the models. This can be done to a certain level by making the parameters of the gene network to depend on those conditions so that the full potential of the chaotic behavior inside the cell can allow the gene network to evolve with the changes of external conditions. From a theoretical point of view, this requires to explicitly change the number of parameters needed to implement such a dependence if the number of external parameters changes (for instance a new molecular gradient or new possible inter-cells interactions appears in the vicinity of the cell) and is then an ill-defined procedure. Moreover, keeping the gene network behavior to be the underlying explanation for cell state switches requires its associated set of differential equations to have non-linearities. Indeed, they are the necessary conditions to have a possible chaotic switch between different final states. This is in our opinion putting a too strong constraint on the possible models of cell differentiation (Villani et al., 2011). In particular, this approach ignores the possible role of the external environment changes on the cell fate and forgets about very important scientific facts such as the stochasticity coming from the local molecular behavior that can be moderated by the organization of the cell, especially of its chromatin. While being partially captured by stochastic gene networks, the intrinsic stochasticity of molecular processes allowing the cell to explore different configurations requires to go beyond genetic networks that only provide a finite number of possible states, while biological systems need to escape this limitation to cope with the full diversity of external conditions.

On the other hand, there is a real physical motivation to assume that such an intrinsic stochasticity inside cells can come either from a fundamental reason related to quantum mechanics when atom/molecular scale processes occur (proton/ion pumps, chromatin binding…) or from temperature effects which imply a distribution of configuration for the various components in the cell. Including such considerations in the model building allows us to offer a much simpler situation where differential equation systems do not need to be non-linear as soon as this intrinsic stochasticity is able to trigger switches between a large variability of cellular states with a related diversity of patterns of gene expression. From a statistical physics point of view, this stochasticity accounts for the fluctuations allowed by the limited number of components involved at the molecular level in cell mechanisms.

**FIGURE 2:**
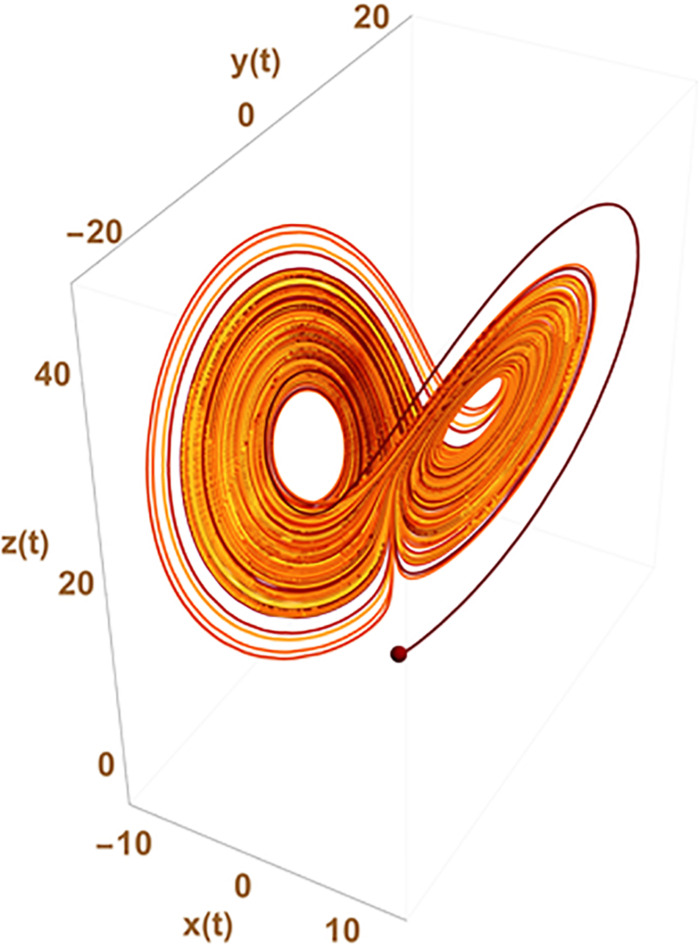
Chaotic trajectories from the historical Lorentz problem. The figure depicts the possibility that states {x,y,z} of the system change apparently randomly from one to the other two well defined regions of the parameter space depicting what has given its name to the “butterfly effect” in chaos theory. The trajectory of the system in the state space switches randomly from trajectories cycling around one of the two strange attractors existing in the ODE system: $\left\{ \begin{array}{c} \frac{d\,x}{d\,t}=-3\,\left(x\,\left(t\right)-y\,\left(t\right)\right),\\ \frac{d\,y}{d\,t}=-x\,\left(t\right)\times z\,\left(t\right)+26.5\,x\,\left(t\right)-y\,\left(t\right),\\ \frac{d\,z}{d\,t}=x\,\left(t\right)\times y\,\left(t\right)-z\,\left(t\right), \end{array}\right.$ with initial conditions x(0) = y(0) = 0 and z(0) = 1.

Such an approach can circumvent the need of chaos to explain statistical variation at the gene expression levels which is usually the expected paradigm from usual differential equation formalism (ODE) used to represent the underlying gene network. In chaos theory, the observed apparent stochasticity occurs from the possible switch of cell trajectory in the cell state space from one strange attractor to another (a point in the configuration space around which the system trajectory is attracted). The number of attractors and their location in the configuration space result from the inner properties of the set of differential equations used to describe the system evolution. This is sketched in Figure 2 where x(t), y(t), and z(t) could, for instance, represent the level of expression of 3 genes giving rise to a variety of observed changes in the cellular state {x,y,z} with time.

The aggregation of the two basic ideas that there is a well physically motivated intrinsic stochasticity inside the cell and that a theoretical approach is needed to include external constraints on the dynamics of biological systems allow to propose a very interesting framework to address biological organization, evolution and emergence of new biological functions in the same theoretical paradigm. Such an approach was first introduced in the theory of ontophylogenesis (Kupiec, 1996, 1997, 2009) in which dynamics takes a major role while usual genetic information still finds a natural place becoming only an important constraint on the dynamics occurring inside the cell. This idea can be extended to also include other structures inside the cells (compartments, large scale molecular machinery…) and in the organism (organs, vascularization…) on the same basis (Laforge, 2009). From the physical point of view, this approach allows us to overtake the standard statistical approaches where the key concept used as a proxy for the information is entropy. This latter physical quantity provides in fact a change of the information concept as usually understood and discussed by biologists and of the associated determinism that is usually invoked to speak about the cell fate in terms of gene expression patterns. Indeed, as discussed a bit earlier, entropy, as a statistical physics concept developed to explain the time’s arrow in statistical systems, expresses the level of order inside a physical system (Blum, 1968). Nevertheless, one should notice that entropy does not provide an adequate way to describe the evolution of organized dissipative structures. Indeed, from a statistical physics point of view, the system is always a set of microscopic components while its mesoscopic or macroscopic description requires the introduction of a mathematical object able to account for this scale of organization.

### Emergence as the Result of a Competition Between Internal Dynamics and External Dynamical Constraints

The second principle of thermodynamics states that a higher order in a structure cannot be obtained without an exchange of entropy with the outside of the system. This means that building models in which a switch in the organization could only come from an internal reorganization is difficult to support. It directly means that using a system of ODE, the only possible hope is to describe a switch between two states with the same level of order at most. This approach is then unsuitable to describe growing structures and organization inside a cell unless something more is added to take into account these entropy exchanges. Importantly, this argument states that some constraints on the organization process of a biological system are arising from physical limitations to energy and matter fluxes exchanged between the cell and its local environment. This argument holds at any scale and also gives inputs to understand how inner cell structures evolve such as chromatin, cell compartments or even proteins during the macromolecular processes involved in gene expression. In these various cases, one envisages different structures with different outsides which show that there is a continuity of scales that need to be considered to understand how biological systems get structured macroscopically. Indeed speaking for instance about chromatin requires to consider cell compartmentation and nuclear membrane in eukaryotic cells as structures that act on the possible fate of chromatin.

The second principle of thermodynamics is coherent with the ontophylogenesis framework explanation of stochasticity control inside a cell as a result of changes in its local environment, including cellular exchanges with its neighbors. It also puts an important light on how emergence of novelty can occur in biology giving a very strong input to the Darwinian theory (Laforge, 2009). Ontophylogenesis, by pushing Darwinian logic inside the cell, i.e., by introducing a random turn-over of different primed states resulting from intrinsic stochasticity of physico-chemical reactions at low scale, provides a key reason to explain the divergence of species seen as a successive collections of individuals intrinsically subject to variations possibly aggregated in seminal cells affecting their whole descendance (Kupiec, 2009). Interestingly, this framework allows external causes to imply a change of an individual’s organization as a special case of constraints on the organism dynamics during its development.

**FIGURE 3:**
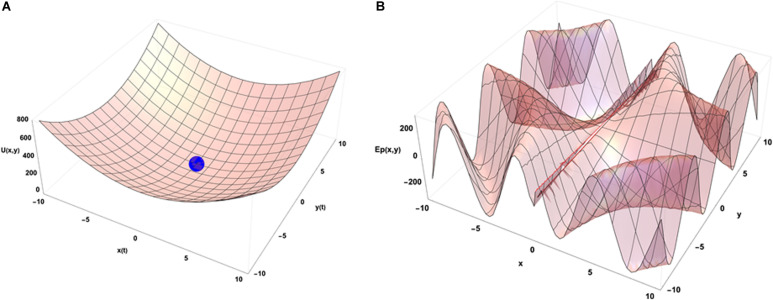
Energetic point of view of the dynamical organization of a system with 2 degrees of freedom in interaction with its environment. **(A)** Internal energy landscape of a system as a function of two variables measuring its geometrical organization. The final configuration of the system is (0,0) without external constraints. **(B)** A potential energy landscape describing the interactions of the system with its external environment as a function of its location in the (x,y) configuration space (*t* = 0). The functional form used to draw that illustrative example is U(x,y) = 4 (x2+y2) for **(A)** and Ep(x,y) = 300 × Sin(0.1 × y + 3 t) × Cos[1/(x + 0.1) + 3 t] for **(B)**. In the latter case, at dependence of the external potential is used to illustrate the impact of external potential energy dynamics on the final organization of the system (see text).

An idea of how emergence occurs in biological systems (and in complex systems in general) can be depicted using the physical concept of internal energy of a system that describes the kinetic energies of its components and the potential energy arising from their geometric organization. In the case of the chromatin system, this corresponds mostly to all the binding energies of molecular interactions that organize its geometrical configuration and to the kinetic energies of the related molecules that have an average speed determined by the temperature inside the cell. Figure 3A illustrates a very simple case in which the internal energy of the system is represented as a function of two conformation variables (x and y).

**FIGURE 4:**
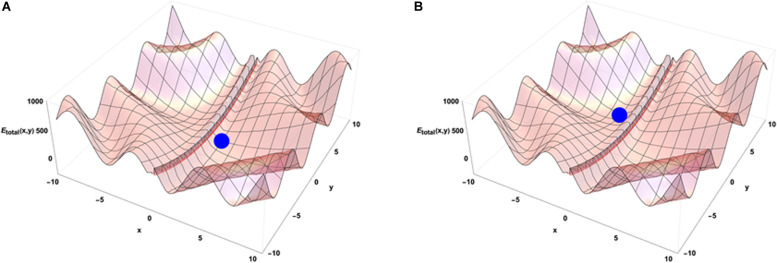
Emergence from the competition of dynamics of external and internal constraints. The subfigures **(A,B)** show that different minima of energy can be reached by the system in its time evolution. The point reached at the same time step of the simulation depends on the position of the system in its initial state and on the evolution dynamics of the external potential.

When the system is isolated, i.e., is not experiencing any external constraints, general physical variational principles state that the system evolves toward the configuration giving a minimum of its internal energy. The configuration (x = 0, y = 0) occupied by the blue point is then depicting the equilibrium position of the system in the energetic landscape. If the system is now put in an environment that is acting on it through external forces, the situation requires to add an external potential energy like the one shown on Figure 3B. In that case, the physics drives the systems so that it minimizes the sum of the two potential energies. Then the final state is not given by the minimal internal energy configuration of the system only but is obtained as a mixed result between the internal and external potentials configuration. Translated to a biological case, if one looks at the differentiation of cells in different conditions of pressure, consequences from those conditions are expected on the cell differentiation as it has been demonstrated *in vivo* (Farge, 2003). Analogously, an impact of external local forces acting on cell membrane is expected on the cell division axis as it has been shown *in vitro* (Thery and Bornens, 2006). Figure 3 illustrates that different final states can be obtained in the evolution of the system if its initial state starts from different positions in the configuration space since there are different minima in the energetic landscape build as the sum of the two potentials. Then the final state depends from the starting point of the system in the energetic landscape. If we now consider that there is a dynamical process ongoing with specific times of evolution for both internal and external potentials, the end point of the evolution depends on the competition between the dynamics of internal and external constraints which is the precise case in which Figures 4A,B have been obtained (the blue point is the position of the system in its state space (x,y) after the same amount of time in the two different simulations) from different initial states under the same dynamics of the external potential. The figures show the sum of the two potential energies at the final time of the simulation. At each step in time, the simulation moves the state of the system toward the closest local minimum around the current position of the system with a speed that is representative to the characteristic time of evolution of the inner dynamics of the system. To illustrate the situation, one can argue that the typical time of evolution of the internal energy is related to the inertia of the components of the system and to the average duration of bounded states inside the system. The external dynamics is characterized by a typical time of external potential energy variations. The meaning of the results of this simulation is that, when one looks at an organized system (one only accesses to the final state of the evolution!), there is no hope to understand its structure from the properties of its local components since we have lost the history of the changes that occurred in its vicinity while it was being structured. The real situation is even a bit more complex if one considers that the matter exchanged between the system and its external world changes deeply the inner potential. This can be seen as an extra internal dynamic of the inner potential allowing us to think about this issue in the heuristic approach proposed here.

This simple example can be extended to address the case for an open system where energy and matter is being exchanged with its external environment where the general situation explained above remains. Rephrased in the language of statistical physics, it seems that one cannot just address the organization of a complex system from its inner components properties only. Indeed, dissipative structures put emergent constraints of the dynamics of the microscopic components. One should also notice that from a theoretical point of view, one needs to use a mathematical structure to describe and follow these mesoscopic or macroscopic organizations in the system. Indeed, it is not enough to just define the evolution of the system of interest by accounting for the amount of entropy it exchanged with the external world. One needs to indicate how the new possible ordering power given by the decrease of the entropy of the system due to this exchange with its external world is used inside the system. This means that the traditional statistical physics approach is ill-defined to address the question of complex system organization with spatial and temporal heterogeneity. Such systems require much more than the phase transition paradigm to be understood. It also means that numerical simulations are, in the context of our current knowledge, the only tool to face these kinds of problems but they also require the definition of what one calls a mesoscopic scale organized structure. Indeed, from the point of view of the microscopic components of the system, any organized structure is just a simple configuration of its parts. The fact that such a structure holds in time is not a property of the local components but comes from the interactions at different scales between mesoscopic structures (Marsland and England, 2018).

One important remark along that approach is that it gives a natural explanation to the apparition of levels in biological systems. Indeed, those levels are effective structures (by opposition to fundamental or ontologic) formed from very long lifetime and precise characteristic size dynamic structures. Ontophylogenesis hence provides a reasonable scientific framework to think about emergent properties in biological systems.

The ontophylogenesis paradigm has been tested using numerical simulations (Laforge et al., 2005) to demonstrate its ability to provide good properties of organization during a developmental process. In that study, it was demonstrated using a very simple model with 2 cell types and a mechanism of cell-to-cell signaling based on physics principles that the ontophylogenesis framework can provide reproducible emergent properties relevant to a developmental process. In particular, *in silico* simulations exhibited the formation of a bilayer of cells whose structure completely results from an equilibrium between the numerical values of the parameters of the simulation and from the choice of the simulation rules conditioning how the local environment of each cell stabilizes its chance of changing its phenotype. Importantly, the simulations showed that the emergent bilayer can reach a stabilized form corresponding to an end of the developmental process without an “end of growth” gene. In that simulation, the emergence of the bilayer is associated with a reduction of the variance of expression patterns of every single cell inside the bilayer. Even more interesting, this work presented further results suggesting that cancer could be a problem of tissue disorganization rather than an initial mutation inside a cancer cell. This is supported by theoretical considerations and experimental evidence over cancer initiation at the tissue level (Capp, 2005, 2017). In a subsequent work (Glisse, 2009), it was demonstrated that a sizable amount of stochasticity in gene expression can provide a quicker and more effective way of building the bilayer structure than a very precisely driven expression. Furthermore, this work also demonstrated that the introduction of apoptosis mechanism can provide a more robust formation of the bilayer structure giving a Darwinian explanation to apoptosis inside multicellular organisms.

The present discussion allows a revisit of the Waddington landscape paradigm often invoked in cell state evolution in the context of noise as discussed previously. In the ontophylogenesis framework, that landscape is dynamic because of the evolution of the external constraints applying to the dynamical system under study. In more biological words, this landscape is dynamic because of the dynamics of the selection conditions that occur in the vicinity of the biological system. On the other hand, the system owns an intrinsic capability to explore the landscape which is a consequence of the stochastic nature of its dynamics. This allows the system to change its route in the landscape with low probability of switching from one valley to the other if the valleys are deep. Cell reprogramming to adapt to new conditions is then a natural process inside the ontophylogenesis framework. The organization of biological systems is then a hetero-organization where the intrinsic stochastic dynamics of the fundamental ingredients of the system and the retroaction of organized structures (seen as dynamical states of equilibrium with a certain time of life) on the local dynamics of the parts have a shared causality in the determination of the fate of the system. In some sense, this framework reconciles both Darwinism (intrinsic divergence) and a form of neo-Lamarckism (action of the environment to guide the evolution of a single individual development but without finalism) as two faces of the same underlying coherent framework to think biology.

## Conclusion

Both biological and physical considerations highlight the need to reconsider developmental processes in the light of the inherent stochasticity of cellular behavior. Considering its origin at the gene expression level, whose stochasticity appears to be modulated by environmental constraints during development, leads to put it forward when wanting to build a coherent framework that would no more try to accommodate classical deterministic or semi-deterministic views with this biological randomness. Being supported by its coherence with long-accepted conceptions in physics, a theory such as ontophylogenesis has the necessary requirements to include both the demonstrated probabilistic behavior at the cellular level, and the stabilizing environmental influence on the ongoing processes by coupling their concomitant actions in the emergence of structured tissues. Adopting such an alternative viewpoint would render developmental biology closer to modern physics, and make biologists enter a new era full of innovative research avenues. It would in particular give a very strong case to further develop simulation approaches of biological systems based on multiagent formalism where interactions at low scale can be input in the model while heterogeneous mesoscopic or macroscopic structures retroaction on the evolution of the simulated biological system are dynamically provided by the simulated geometrical organization of the microscopic components of the system. As initial structures result from the previous evolution of the system under a dynamics of external constraints that cannot be known from the observation of the structures themselves, initial state of the simulation should be set from precisely measured structures observed in the real systems that the simulation is to be compared with.

Importantly, ontophylogenesis allows cell differentiation, development and evolution to be thought in the same framework as the cumulative evolution of germinal cells inside successive individuals. Its proposal to see deterministic processes as stochastic processes with a probability of one, as the results of the constraints on the system dynamics, accommodates the known mainly deterministic properties of biological systems. Complementary, its ability to predict the restriction of stochasticity in the process of cell differentiation (Laforge et al., 2005) which has been experimentally observed afterward (Moussy et al., 2017) validates its nature of scientific theory.

## Data Availability Statement

The original contributions presented in the study are included in the article, further inquiries can be directed to the corresponding author.

## Author Contributions

J-PC and BL formulated the hypotheses and wrote the manuscript.

## Conflict of Interest

The authors declare that the research was conducted in the absence of any commercial or financial relationships that could be construed as a potential conflict of interest.
